# Supplementary material for: Liquid Biopsy in the Oncological Management of a Histologically Undiagnosed Lung Carcinoma: A Case Report
Source: J Pers Med. 2022 Nov 9;12(11):1874. doi: 10.3390/jpm12111874 (PMC9694216; doi:10.3390/jpm12111874)
Supplement: Supplementary file 1 [file jpm-12-01874-s001.zip › jpm-1872673-supplementary.pdf]

Supplementary Figure S1.

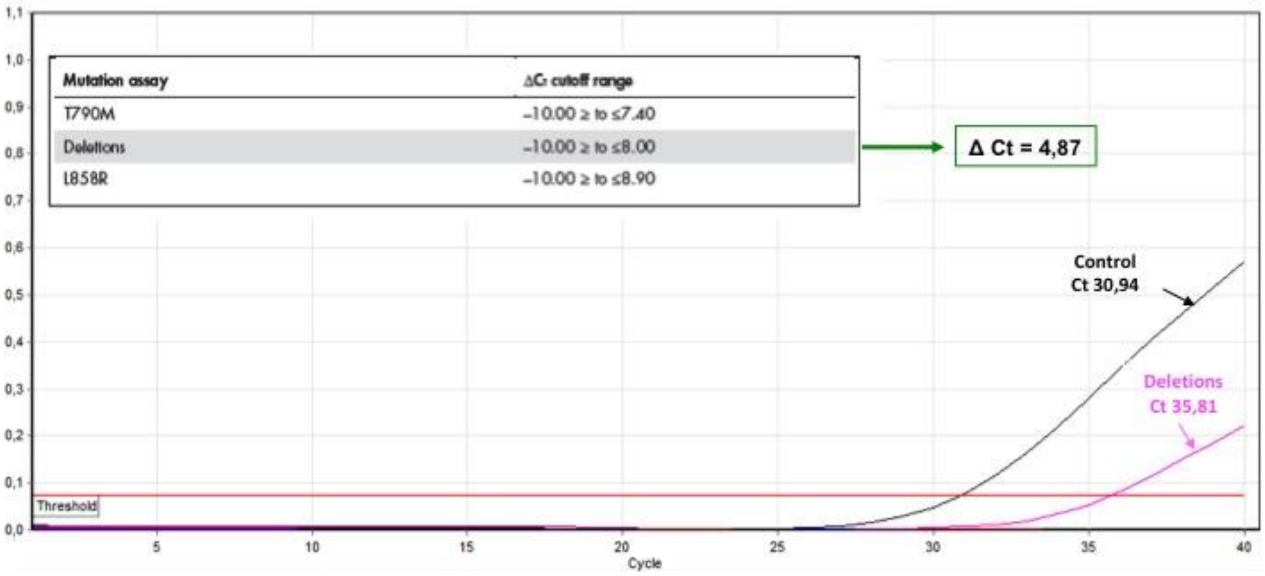

Supplementary Figure S1. Real-time PCR curve of the EGFR mutation analysis on baseline cfDNA. The positive PCR control (black curve) and the mutation signal (pink curve) are reported. Δ Ct, difference in Ct measures - as determined by the number of PCR cycles - between mutated and control samples.
